# Expression of CD11c Is Associated with Unconventional Activated T Cell Subsets with High Migratory Potential

**DOI:** 10.1371/journal.pone.0154253

**Published:** 2016-04-27

**Authors:** Jamal Qualai, Lin-Xi Li, Jon Cantero, Antoni Tarrats, Marco Antonio Fernández, Lauro Sumoy, Annie Rodolosse, Stephen J. McSorley, Meritxell Genescà

**Affiliations:** 1 Mucosal Immunology Unit, Institut d’Investigació en Ciències de la Salut Germans Trias i Pujol, AIDS Research Institute IrsiCaixa-HIVACAT, Can Ruti Campus, Badalona, Spain; 2 Department of Microbiology and Immunology, University of Arkansas for Medical Sciences, Little Rock, Arkansas, United States of America; 3 Department of Obstetrics and Gynecology, University Hospital “Germans Trias i Pujol,” Can Ruti Campus, Badalona, Spain; 4 Flow Cytometry Unit, Institut d’Investigació en Ciències de la Salut Germans Trias i Pujol, Badalona, Spain; 5 Genomics and Bioinformatics Group, Institute for Predictive and Personalized Medicine of Cancer (IMPPC), Can Ruti Campus, Badalona, Spain; 6 Functional Genomics Core, Institute for Research in Biomedicine (IRB Barcelona), Barcelona, Spain; 7 Center for Comparative Medicine, Department of Anatomy, Physiology and Cell Biology, School of Veterinary Medicine, University of California, Davis, Davis, California, United States of America; Harvard Medical School, UNITED STATES

## Abstract

CD11c is an α integrin classically employed to define myeloid dendritic cells. Although there is little information about CD11c expression on human T cells, mouse models have shown an association of CD11c expression with functionally relevant T cell subsets. In the context of genital tract infection, we have previously observed increased expression of CD11c in circulating T cells from mice and women. Microarray analyses of activated effector T cells expressing CD11c derived from naïve mice demonstrated enrichment for natural killer (NK) associated genes. Here we find that murine CD11c^+^ T cells analyzed by flow cytometry display markers associated with non-conventional T cell subsets, including γδ T cells and invariant natural killer T (iNKT) cells. However, in women, only γδ T cells and CD8^+^ T cells were enriched within the CD11c fraction of blood and cervical tissue. These CD11c^+^ cells were highly activated and had greater interferon (IFN)-γ secretory capacity than CD11c^-^ T cells. Furthermore, circulating CD11c^+^ T cells were associated with the expression of multiple adhesion molecules in women, suggesting that these cells have high tissue homing potential. These data suggest that CD11c expression distinguishes a population of circulating T cells during bacterial infection with innate capacity and mucosal homing potential.

## Introduction

The ability to assess ongoing mucosal immune responses is critical for understanding host-pathogen responses and would assist mucosal vaccine development. In the case of the female genital tract (GT) immune responses, assays to determine the magnitude and quality of the immune response in mucosal tissues largely rely on sampling of peripheral blood. Measuring antigen-specific T cell responses directly is not always technically or economically feasible, therefore methods that provide an indirect measure of the immune response are essential.

We previously detected an increase in the expression of α chain integrin (αX, CD11c molecule) when analyzing blood samples from mice with lower GT *Chlamydia* infection and women with symptomatic bacterial vaginosis *(J*. *Qualai et al*., *submitted for publication)*. Integrins are widely expressed cell surface molecules, composed of non-covalently linked α and β subunits that allow cell-extracellular matrix and cell-cell interactions [1]. Among these integrins, CD11c/CD18 is one of the four members of the β2 leukocyte integrin family [1]. CD11c is also referred to as complement receptor 4, since it can mediate phagocytosis of inactivated complement C3b-opsonized particles, demonstrating a wider role in host defense than cell-cell adhesion [2]. There are a wide variety of ligands described for CD11c, including other adhesion molecules, bacterial cell wall components (including lipopolysaccharide), complement proteins, and matrix proteins [2]. The CD11c/CD18 complex is also reported to bind denatured proteins, perhaps acting as a danger signal in the context of innate immune defense [3].

CD11c is typically considered to be a marker of conventional dendritic cells (DC) [4], and is not often considered in the context of T cell responses [5]. Yet, CD11c can be expressed on NK cells and populations of activated T and B cells [6]. Indeed, studies in several different mouse models have associated CD11c expression with effector memory and regulatory T cell subsets (T_EM_/T_regs_) [7–12], small gut intra-epithelial lymphocytes [13, 14], and the development of experimental autoimmune encephalomyelitis [15]. The exact role of CD11c is unclear but was found to correlate with increased cytotoxicity, effector, or regulatory function (*reviewed in* [16]). CD11c expression has also been associated with activated antigen-specific T cells expanded in response to infection or vaccination, and has been correlated with the potential of these cells to secrete IFN-γ [9, 12]. Recent reports have also described a unique cellular subset in mice and humans that contains key features of both T cells and DCs [17]. Interestingly, these unusual cells are characterized by the expression of T cell receptor (TCR), major histocompatibility complex (MHC) II, and CD11c [17].

Our hypothesis is that increased CD11c expression in circulating T cells during bacterial infection of the GT indicates the activation and migration of innate-like T cells. Here, we have examined the phenotype of T cells expressing CD11c in the blood and GT of mice and women. We find that in addition to CD8^+^ T cells, γδ T cells represent an unconventional T cell subset that expresses CD11c under physiological conditions. These innate populations should be considered when evaluating mucosal immune responses to infection.

## Materials and Methods

### Ethics statement

All animal procedures were approved and supervised by the Animal Care Committee of the Germans Trias i Pujol Health Science Research Institute (IGTP) and by the Department of Environment of the Catalan Government (approval number # 7066).

Informed written consent was obtained from all participants and the study protocols and questionnaires were approved by the University Hospital Germans Trias i Pujol (HUGTP, Badalona, Spain) Clinical Research Ethics Committee (reference numbers # EO-11-074 and # PI-14-070). The study was undertaken in accordance with the Declaration of Helsinki and the requirements of Good Clinical Practice.

### Samples for differential gene expression analysis

Six to eight week-old female C57BL/6 mice were bred in house. Mice were kept in ventilated cages with sterile food and water *ad libitum*, under specific-pathogen-free conditions at the animal facility for experimental models of the IGTP, Spain. A group of six females were treated subcutaneously with 3mg of Depo-Provera six days prior to euthanizing in order to homogenize the endocrine effects on immunity by synchronizing the menstrual cycle. All mice were euthanized with isoflurane (inhalation excess) and blood was immediately obtained by cardiac puncture.

Blood samples (~500μl) were immediately lysed, washed, suspended in PBS and incubated with Aqua vital dye to distinguish dead cells (Invitrogen, Burlington, ON, Canada). Cells were suspended in staining buffer (1% BSA-PBS) and incubated with CD3-Vioblue, CD4-APC-H7, CD62L-PE (Miltenyi Biotec, Madrid, Spain), CD44-Brilliant Violet 570 (BioLegend, San Diego, CA) and CD11c-PE-Cy7 (HL3) (BD Biosciences, San Jose, CA). Cells were suspended in cold FACS-flow buffer (1% FBS-PBS with 0.5mM EDTA) and immediately sorted into CD3^+^ CD62L^-^ CD44^high^ CD11c^+^ and CD3^+^ CD62L^-^CD44^high^ CD11c^-^ using a BD FACSAria^TM^ Cell Sorter. Purity of sorted cells was >99%. Each sample was sorted by collecting 300 cells directly into refrigerated lysis buffer [18], immediately spun, heated at 65°C for 15 minutes and kept at 4°C until delivery to the Institute for Research in Biomedicine (IRB, Barcelona, Spain).

### Differential gene expression and gene set enrichment analysis

Microarray gene expression profiling with Mouse Genome 430 PM Strip arrays was performed following manufacturer recommendations (Affymetrix Inc, Santa Clara, CA) with specific adaptations based on the picoprofiling method [18]. Image intensities were extracted with Affymetrix GeneAtlas System software, normalized and summarized by RMA and analyzed for differential gene expression by Limma [19] with false discovery rate multiple testing significance correction. NK-related γδTCR, iNKT CD4^+^ and iNKT CD4^-^ cells specific gene sets [20] were tested by Gene Set Enrichment Analysis (GSEA) [21] for significant enrichment in CD11c^+^ T cells.

### Animal model

*Chlamydia muridarum* elementary bodies were purified by discontinuous density gradient centrifugation and the number of inclusion-forming units were determined as previously described [22, 23]. Eight week-old C57BL/6 mice were purchased from The Jackson Laboratory (Bar Harbor, ME). All mice were maintained in accordance with University of California Davis Research Animal Resource guidelines, USA. Estrus was synchronized and 7 days after, 1x10^5^ C. muridarum in 5μL sucrose/phosphate/glutamate buffer were deposited directly into the vaginal vaults with a blunted pipet tip [23].

### Tissue processing

Blood was collected by retro-orbital bleeding and erythrocytes were immediately removed by ACK lysing buffer (Life Technologies). Leukocytes were washed with FACS buffer (PBS with 2% FBS) and stored on ice until use. Mouse GT were removed and leukocytes isolated as described [24]. Briefly, vagina, cervix, uterine horns and oviducts were minced into small pieces, digested in 500mg/L collagenase IV (Sigma) for 1 hour at 37˚C with constant stirring. Leukocytes were purified by percoll density gradient centrifugation (GE Healthcare), washed with FACS buffer and stored on ice until use.

### T cell subset analyses in mouse tissues

Leukocytes from blood and GT were harvested from naïve or infected mice as described above. Single cell suspensions were prepared in FACS buffer and blocked with Fc block (culture supernatant from the 24G2 hybridoma, 2% mouse serum, 2% rat serum and 0.01% sodium azide). Cells were stained with different panels of antibodies including: FITC-CD4 (GK1.5), CD8 (53–6.7), NKp46 (29A1.4; Biolegend), PE-CD49b (DX5), CD103 (2E7), NK1.1 (PK136), γδTCR (eBioGL3), PerCP-Cy5.5-CD11c (N418), Alexa Fluor 700-CD3 (eBio500A2), eFluor 450-CD8 (53–6.7), NK1.1; APC-CD19 (MB19-1), CD159a (16A11) (Biolegend), CCR10 (248918; R&D Systems, MN) and APC labeled mCD1d PBS-57 tetramer (NIH Tetramer Core Facility). All antibodies were obtained from eBiosciences (San Diego, CA) unless otherwise noted. Flow data was acquired on an LSRFortessa flow cytometer and analyzed using FlowJo vX.0.7 software (Tree Star, Ashland, OR). Because of the low number of CD3^+^ events in the GT of naïve animals, we analyzed some of the frequencies as % of the total number of events (i.e. CD3^+^ or CD3^+^ CD11c^+^); while others, like the expression of certain molecules within the CD3^+^ CD11c^+^/^-^ fractions, were analyzed as the % of the parent as we did for blood (i.e. CD3^+^ CD11c^+^/^-^ TCRγδ^+^). Boolean gating strategy was used for simultaneous expression of different molecules, which were represented visually using Pestle (v1.7) and Spice (v5.3) softwares (provided by the National Institutes of Health) [25].

### Phenotyping of human blood

Healthy normal donors (ND) were recruited from the clinical trials unit of the HUGTP. For unconventional T cell subset phenotyping (n = 10, 27±4 years old), 1ml of blood was lysed by ammonium Lysis Buffer (BD Pharm Lyse, BD Biosciences) and processed for staining. Leftover blood (∼9ml) was used to isolate peripheral blood mononuclear cells (PBMC) by density gradient centrifugation over Ficoll gradient (Biochrom AG, Berlin, Germany). Both cell suspensions were stained with: CD3-eFluor 605 (OKT3, eBiosciences), CD14-V450 (MØP9), CD19-V450 (HIB19), CD11c-PE-Cy7 (B-ly6), CCR7-Horizon PE-CF594 (150503), CD8-V500 (RPA-T8) (BD Biosciences), NK1.1-PE (191B8), γδTCR-FITC (11F2), Vα7.2-APC-H7 (REA179) (Miltenyi Biotec) and Vα24-APC (6B11) (BioLegend).

For adhesion molecules phenotyping, samples (n = 13, 26±3 years old) were analyzed as described *(J*. *Qualai et al*., *submitted for publication)*. Briefly, after erythrocyte depletion from 1ml of blood, cells were stained with Aqua Dye (Invitrogen), washed again, suspended in staining buffer and divided into four tubes. Common antibodies to each panel were: CD3-eFluor 605, CD4-Alexa700 (eBioscience), CCR7-Horizon PE-CF594, CD38-Brillant Violet 421, HLA-DR-PerCP-Cy5.5 and CD11c-PE-Cy7 (BD Biosciences). Specific for each panel were: 1) CCR2-PE, CCR5-APC-Cy7, CXCR6-APC (R&D Systems Inc.) and CXCR3-FITC (BioLegend); 2) CD49d (α4)-FITC, β7-APC, CCR9-PE (BD Biosciences) and CD29 (β1)–APC-Cy7 (BioLegend); 3) CD103-FITC, CD54-APC, CD49a (α1)-PE and CD29 (β1)–APC-Cy7 (all from BioLegend); 4) CD18-APC, CLA-FITC (both from BD Biosciences) and CCR10-PE (BioLegend). For both analyses cells were acquired using a BD LSRFortessa SORP flow cytometer (Flow Cytometry Platform, IGTP) and analyzed with FlowJo vX.0.7 software (TreeStar). Gates were drawn based on fluorescence minus one-controls.

### Cervical tissue digestion and flow cytometry

Human cervical tissue was obtained from two sets of five healthy women (age range 42–47 years old) undergoing hysterectomy for benign indication at HUGTP. After confirmation of healthy tissue status by the Pathological Anatomy Service, a piece from ecto and endocervix separated by anatomical localization was delivered to the laboratory in refrigerated RPMI 1640 medium (Cellgro, Manassas, VA) containing 10% FBS (Lonza, Basel, Switzerland), 500U/mL penicillin, 500μg/mL streptomycin, 5μg/mL fungizone and 1μg/mL gentamycin (Life Technologies). Tissue was processed within the next 12 h after surgery, and 8-mm^3^ block-dissection was performed as described [26]. Tissue digestion of 5–9 pieces of ecto or endocervix with collagenase IV (Invitrogen) was immediately executed as described [26]. Tissue blocks were then dissociated manually with a disposable pellet pestle and filtered through a 70μm-cell strainer (BD Biosciences). After centrifugation, pellet was suspended in staining buffer (1% mouse serum, 1% goat serum in PBS) and stained with different combinations of CD3-eFluor 605 (eBiosciences), CD14-V450, CD11c-PE-Cy7, CD8-V500, HLA-DR-PerCP-Cy5.5, CD69-Horizon PE-CF594 (BD Biosciences), NK1.1-PE, γδTCR-FITC, Vα7.2-APC-H7 (Miltenyi Biotec), CD103-FITC and Vα24-APC (BioLegend). Data were acquired and analyzed as described for blood.

### PBMC activation and intracellular cell staining

PBMC were cultured in RPMI 1640 supplemented with 10% FBS and 40μg/ml gentamycin. Stimulation was performed with 1μl (E)-4-hydroxy-3-methyl-but-2-enyl pyrophosphate (HMBPP, Sigma). After 18 h of activation, Brefeldin A (GolgiPlug, BD Biosciences) was added. Five hours later, cells were stained with CD3-PerCP (OKT3), CD11c-PE-Cy7 (B-ly6), HLA-DR-PerCP-Cy5.5 (G46-6), CD69-Horizon PE-CF594 (FN50) (BD Biosciences) and γδTCR-PE (11F2) (Miltenyi Biotec). After surface staining cells were fixed and permeabilized with Fix/Perm Kit and intracellularly stained with IFN-γ-Alexa700 (B27) (Invitrogen). Data were acquired and analyzed as described for blood.

### Statistical Analysis

Data are reported as the mean and the standard deviation (SD) for each group using Prism 4.0 software (GraphPad Software). For animal samples, statistical analyses were performed by Student’s t test or by Paired T test when comparing the positive and negative fraction of the same sample. For all the other analyses in human samples, which did not pass normality test (p<0.05), non-parametric tests were employed. A p value of <0.05 was considered significant.

### Accession codes

Microarray data presented in this article are deposited into the Gene Expression Omnibus (http://www.ncbi.nlm.nih.gov/geo/) under accession number GSE68934.

## Results

### Murine activated CD11c^+^ T_EM_ cells are enriched for NK gene signatures

In order to characterize circulating CD11c^+^ T cells, we initially analyzed gene expression in activated CD3^+^ CD62L^-^ CD44^+^ CD11c^+^ and CD3^+^ CD62L^-^ CD44^+^ CD11c^-^ T_EM_ cells recovered from naïve female mice. Multiple related genes were expressed two to eight fold higher in CD11c^+^ than CD11c^-^ T cells (**Table 1**). Many of these genes are expressed in NK and non-conventional memory T cell subsets but are not highly expressed in conventional αβ T cells [20]. Gene Set Enrichment Analysis revealed significant enrichment for γδT, NK and CD4^-^ iNKT cells when looking at the entire dataset (**S1 Fig**). Thus, CD11c expressing circulating T cells display a gene signature similar to γδ T and CD4^-^ iNKT cells.

**Table 1:** Example of up-regulated genes in activated effector CD11c^+^ vs. CD11c^-^ T cells in naive mice.

Gene Symbol	Gene Title	Log Fold Change	Adjusted *p* Value
C3ar1	complement component 3a receptor 1	6.20	0.0081
C5ar1	complement component 5a receptor 1	5.80	0.0132
Ccl3	chemokine (C-C motif) ligand 3	7.04	0.0050
Ccl4	chemokine (C-C motif) ligand 4	5.23	0.0055
Ccl6	chemokine (C-C motif) ligand 6	7.19	0.0055
Ccl9	chemokine (C-C motif) ligand 9	6.39	0.0050
Cd244	CD244 natural killer cell receptor 2B4	3.75	0.0081
Cd8a	CD8 antigen, alpha chain	2.36	0.0490
Csf1	colony stimulating factor 1 (macrophage)	5.98	0.0156
Csf1r	colony stimulating factor 1 receptor	5.95	0.0077
Csf2ra	colony stimulating factor 2 receptor, alpha, low-affinity (granulocyte-macrophage)	4.69	0.0240
Csf2rb	colony stimulating factor 2 receptor, beta, low-affinity (granulocyte-macrophage)	7.74	0.0055
Csf2rb2	colony stimulating factor 2 receptor, beta 2, low-affinity (granulocyte-macrophage)	6.66	0.0055
Csf3r	colony stimulating factor 3 receptor (granulocyte)	2.36	0.0300
Gzma	granzyme A	3.66	0.0055
Gzmb	granzyme B	2.93	0.0490
H2-Aa	histocompatibility 2, class II antigen A, alpha	8.91	0.0050
H2-Ab1	histocompatibility 2, class II antigen A, beta 1	6.86	0.0065
H2-DMa	histocompatibility 2, class II, locus DMa	3.63	0.0063
H2-DMb1 /// H2-DMb2	histocompatibility 2, class II, locus Mb1 /// histocompatibility 2, class II, locus Mb2	4.46	0.0055
H2-DMb2	histocompatibility 2, class II, locus Mb2	4.76	0.0225
H2-Eb1	histocompatibility 2, class II antigen E beta	5.93	0.0121
Ifitm1	interferon induced transmembrane protein 1	6.42	0.0050
Ifitm2 /// LOC631287	interferon induced transmembrane protein 2 /// interferon-induced transmembrane protein	5.09	0.0063
Ifitm3	interferon induced transmembrane protein 3	6.20	0.0276
Klra2	killer cell lectin-like receptor, subfamily A, member 2	3.45	0.0258
Klrb1b	killer cell lectin-like receptor subfamily B member 1B	3.43	0.0253
Tgfbi	transforming growth factor, beta induced	5.69	0.0050
Tlr7	toll-like receptor 7	2.47	0.0450
Tlr9	toll-like receptor 9	2.95	0.0065

### Murine CD11c^+^ T cells express high levels of NK1.1 in blood after vaginal infection

**Fig 1:**
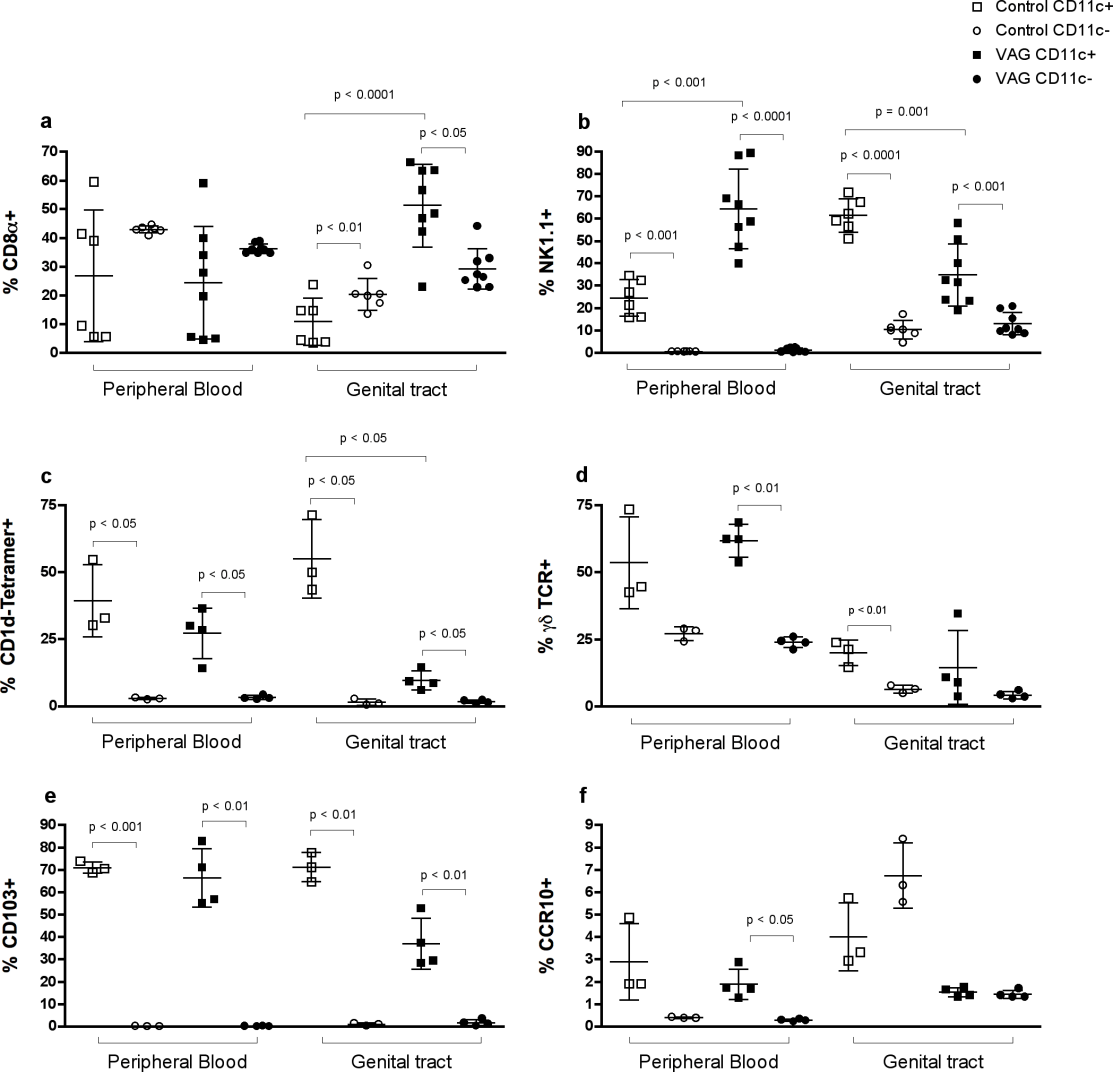
T cell phenotypes in blood and genital tract of mice by CD11c expression. Comparison on the frequency of (**a**) NK1.1, (**b**) CD8α, (**c**) CD1d-tetramer, (**d**) γδTCR, (**e**) CD103 and (**f**) CCR10 in CD11c^+^ (squares) and CD11c^-^ (circles) T cells from the same individual. Gating strategy consisted on a lymphocyte gate based on FSC vs. SSC, followed by doublet exclusion and a CD3^+^ T cells gate. After gating on CD11c^+^ or CD11c^-^ T cells, surface expression of the different markers was quantified (*see* **S2 Fig** *for further details*). Each bar represents the mean ± SD of control (white; n = 3 or n = 6) or vaginally (VAG)-infected mice (black; n = 4 or n = 8) seven days after infection. Data were analyzed using the paired Student’s t-test.

**Fig 2:**
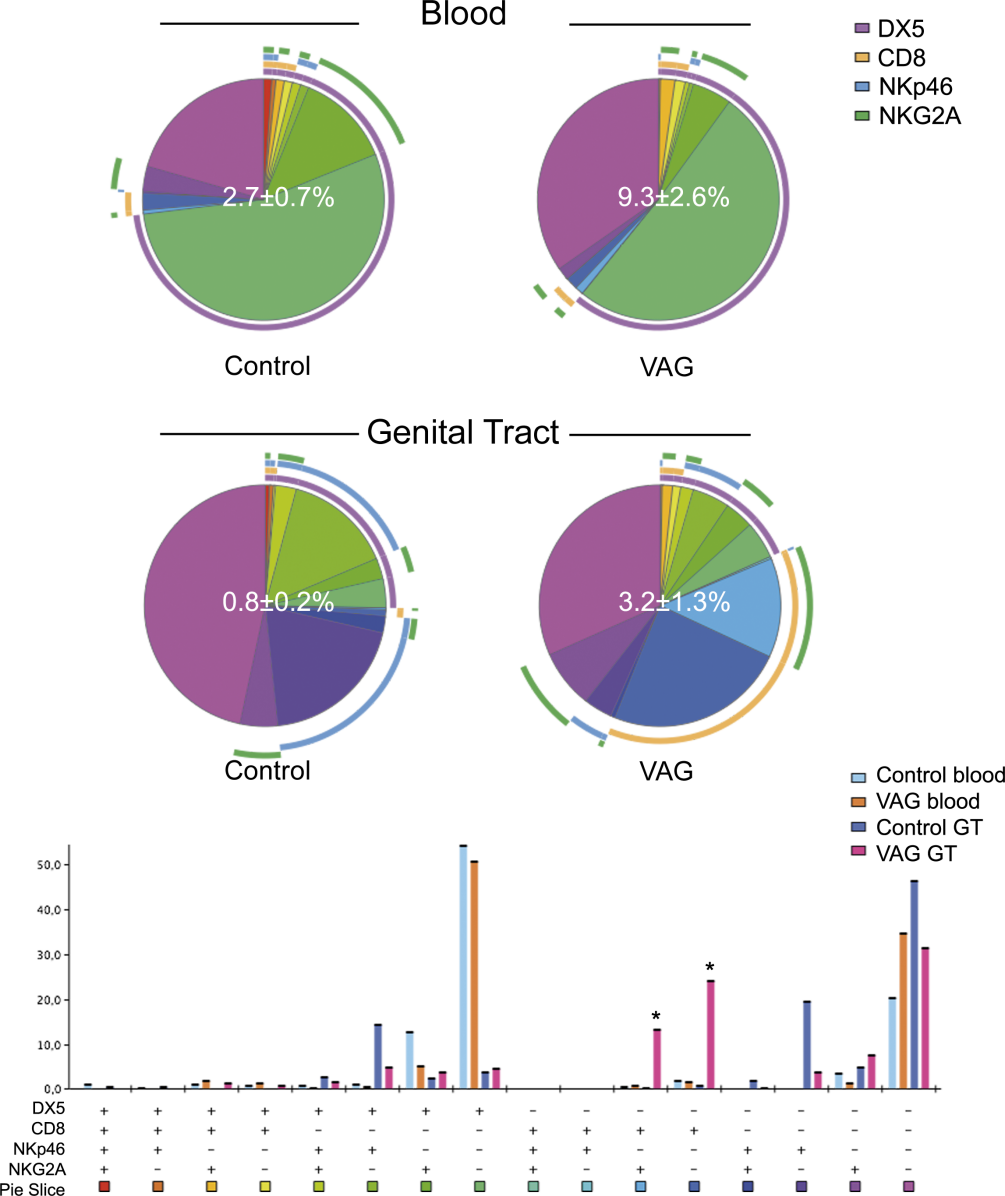
NK phenotypes included in the CD11c^+^ T cell fraction after vaginal Chlamydia infection in mice. The frequency of the different subsets obtained from combining DX5, CD8α, NKp46 and NKG2A expression is displayed for each group as a pie chart and as a complementary bar graph. Gating strategy was performed as described in **Fig 1** and in Materials and Methods. The frequency of CD11c positive cells in the T cell fraction as the mean ± SD is shown as a white number in the center of the pie chart for each group in blood (top) and genital tract (GT, bottom). Each colored portion of a pie chart indicates the percentage of a specific subset detailed in the bar chart below. The arcs around the pie show the molecule or combination of molecules to which those proportions correspond (see color legend indicating DX5, CD8α, NKp46 and NKG2A). *Indicates p<0.05 by Student’s t test analyses only for values >5% of the total CD11c^+^ T cells in vaginally (VAG)-infected mice seven days after infection (n = 4) compared to control (n = 3) animals.

Next, we performed flow cytometry analyses of blood and GT obtained from C57BL/6 mice vaginally infected with *Chlamydia muridarum*. Seven days after infection, the frequency of T cells expressing CD11c^+^ in peripheral blood increased from 1.53% to 6.04% as reported *(J*. *Qualai et al*., *submitted for publication)*. Within this fraction we analyzed the expression of several NK-associated molecules (NK1.1, DX5, NKG2A, NKp46), specific T cell phenotypes (CD8α, CD1d-restricted iNKT cells, γδTCR) and adhesion molecules (CD103, CCR10). Part of the gating strategy is shown in **S2 Fig.** The frequency of CD11c^+^ T cells expressing CD8α in blood was similar before and after infection, while NK1.1 expression increased markedly following infection (**Fig 1A and 1B**). Before infection, a significant fraction of CD3^+^ CD11c^+^ T cells also expressed DX5 and NKG2A (**Fig 2**), but these markers either decreased or remained the same after infection (**Fig 2**).

The frequency of CD3^+^ CD11c^+^ T cells within the GT increased from 0.5±0.2% to 4.1±1.6% seven days after infection (p = 0.0001). In marked contrast to blood, the expression of CD8 by CD11c^+^ T cells strikingly increased in these tissues after *Chlamydia* infection (**Fig 1A**). NK1.1 was expressed at high levels by GT CD11c^+^ T cells before infection and significantly decreased after infection (**Fig 1B**). The expression of DX5 and NKG2A was less frequent at baseline in GT CD11c^+^ T cells compared to blood, while the activating receptor NKp46 was expressed by approximately 40% of cells (**Fig 2**). After infection, the frequency of NKG2A expression by CD11c^+^ T cells significantly increased (p = 0.006), while NKp46 decreased (p = 0.0009) (**Fig 2**). Thus, CD3^+^ CD11c^+^ T cells increase in both blood and GT after vaginal infection and express different patterns of surface markers within these compartments.

### Murine CD11c^+^ T cells include high frequencies of γδT cells and iNKT cells

**Fig 3:**
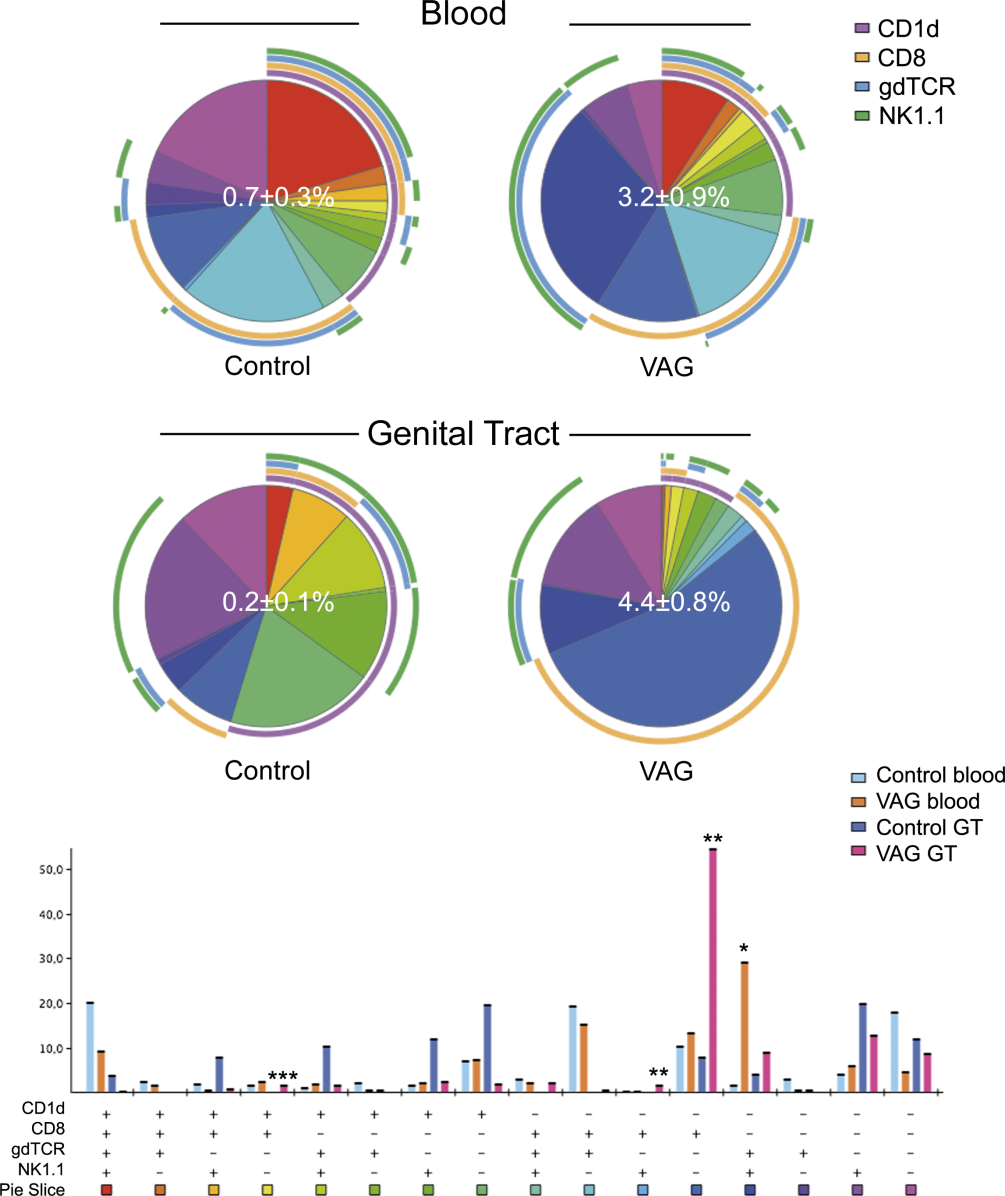
Unconventional phenotypes included in the CD11c^+^ T cell fraction after vaginal Chlamydia infection in mice. The frequency of the different subsets obtained from combining CD1d-tetramer, CD8α, γδTCR and NK1.1 expression is displayed for each group as a pie chart and as a complementary bar graph. Gating strategy was performed as described in **Fig 1** and in Materials and Methods. The frequency of CD11c positive cells in the T cell fraction as the mean ± SD is shown as a white number in the center of the pie chart for each group in blood (top) and genital tract (GT, bottom). Each colored portion of a pie chart indicates the percentage of a specific subset detailed in the bar chart below. The arcs around the pie show the molecule or combination of molecules to which those proportions correspond (see color legend indicating CD1d-tetramer, CD8α, γδTCR and NK1.1). *Indicates p<0.05 by Student’s t test analyses only for values >5% of the total CD11c^+^ T cells in vaginally (VAG)-infected mice seven days after infection (n = 4) compared to control (n = 3) animals.

The total frequency of iNKT or γδ T cells in blood did not change after vaginal infection; however, it did increase in the GT of the infected mice (p<0.003; data not shown). When comparing CD11c^+^ and CD11c^-^ subsets, there was a clear enrichment of these unconventional T cells within the CD11c^+^ fraction in both tissues (**Fig 1C and 1D**). Thus, under physiological conditions, CD11c^+^ T cells included almost exclusively all iNKT cells and high proportions of γδ T cells. Of CD3^+^ CD11c^+^ T cells in the blood, 27–39% of them were iNKT and 50–60% were γδ T cells and this frequency did not change after vaginal infection (**Fig 1C and 1D**). Nevertheless the frequency of CD1d-tetramer^+^ cells in the CD11c^+^ T cell fraction decreased in the GT after infection (**Fig 1C**). Actually, when performing the analysis in combination with CD8 and NK1.1 expression, it was clear that while NK1.1^+^ γδ T cells characterized the major subset expanding in blood, a single CD8^+^ phenotype was the major contributor to the increment of CD11c^+^ T cells observed in the GT (**Fig 3**).

### CD103 expression is exclusively associated to CD11c^+^ T cells in mice

Previous work in a mouse model of colitis determined that CD11c^+^ CD8^+^ T_regs_ are CD8α^+^ CD103^+^ NK1.1^-^ in the small intestine, but CD8α^-^ CD103^-^ NK1.1^+^ in the colon [10]. We analyzed the expression of these molecules and included CCR10, which could indicate migration of CD11c^+^ T cells into the GT. The ligand for this chemokine, CC-chemokine ligand 28, is expressed in the genital tract, and B cells homing there express CCR10 [27]. Co-expression of CD103 and CD11c was high in the blood and GT of uninfected mice (**Fig 1E**). Additionally, among circulating T cells, CCR10 expression was found almost exclusively associated with the CD11c^+^ subset (**Fig 1F**). This chemokine receptor represented a small fraction of CD11c^+^ T cells, but expression was clearly enriched in CD11c^+^ cells after infection (**Fig 1F**).

**Fig 4:**
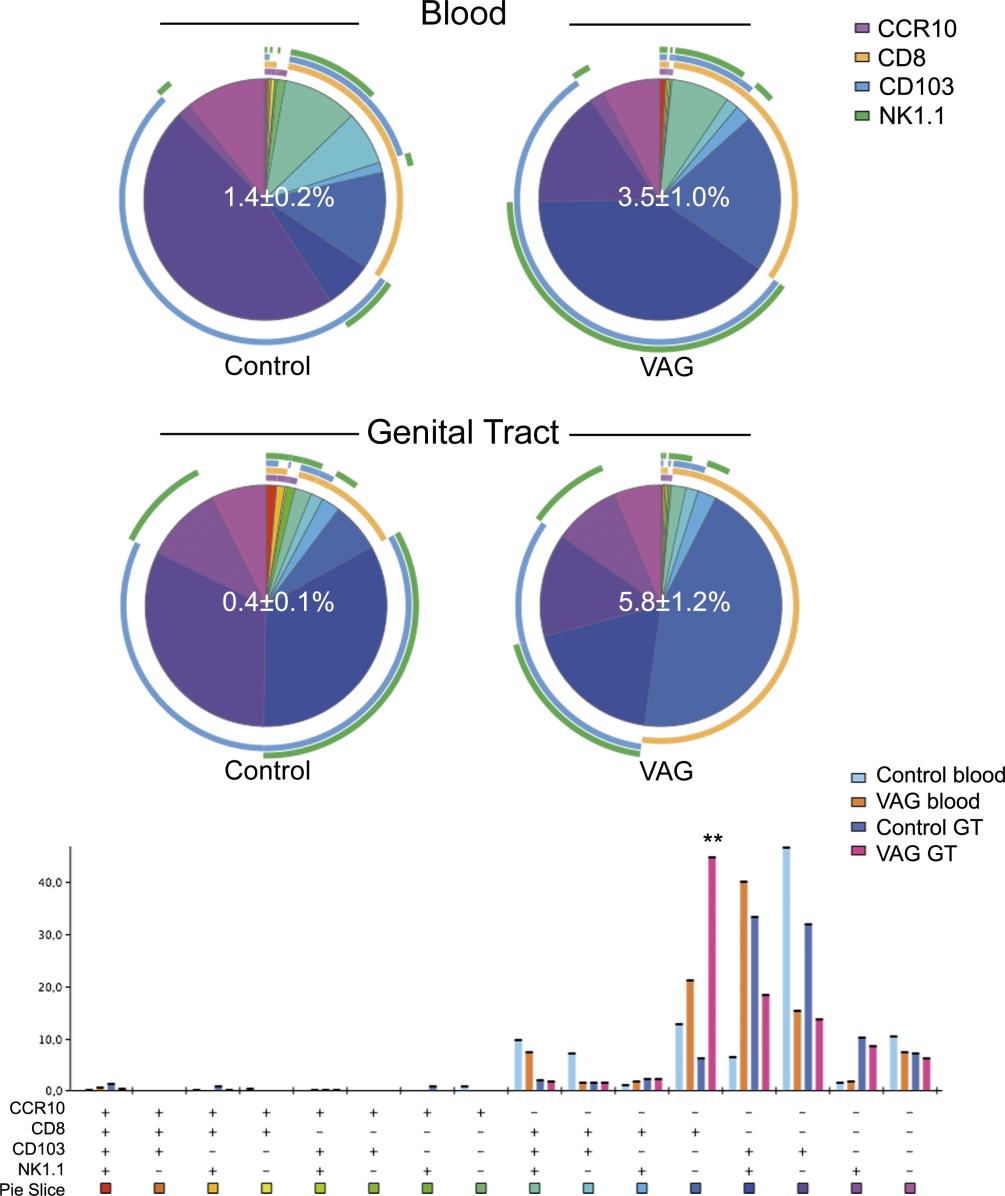
Adhesion molecules included in the CD11c^+^ T cell fraction after vaginal Chlamydia infection in mice. The frequency of the different subsets obtained from combining CCR10, CD8α, CD103 and NK1.1 expression is displayed for each group as a pie chart and as a complementary bar graph. Gating strategy was performed as described in **Fig 1** and in Materials and Methods. The frequency of CD11c positive cells in the T cell fraction as the mean ± SD is shown as a white number in the center of the pie chart for each group in blood (top) and genital tract (GT, bottom). Each colored portion of a pie chart indicates the percentage of a specific subset detailed in the bar chart below. The arcs around the pie show the molecule or combination of molecules to which those proportions correspond (see color legend indicating CCR10, CD8α, CD103 and NK1.1). *Indicates p<0.05 by Student’s t test analyses only for values >5% of the total CD11c^+^ T cells in vaginally (VAG)-infected mice seven days after infection (n = 4) compared to control (n = 3) animals.

To summarize, it appears that the majority of CD11c^+^ T cells in blood under homeostatic conditions are CCR10^-^ CD8α^-^ CD103^+^ NK1.1^-^, but this phenotype is less frequent in the GT (**Fig 4**). After vaginal infection, CD103^+^ NK1.1^+^ CD8α^-^ cells expand among circulating CD11c^+^ T cells and CD8α^+^ CD103^-^ NK1.1^-^ are elevated in the GT (**Fig 4**). Thus, none of these phenotypes illustrated the T_regs_ described in the colitis model [10].

### Circulating T cells expressing CD11c are associated with γδ T cells but not iNKT cells in women

**Fig 5:**
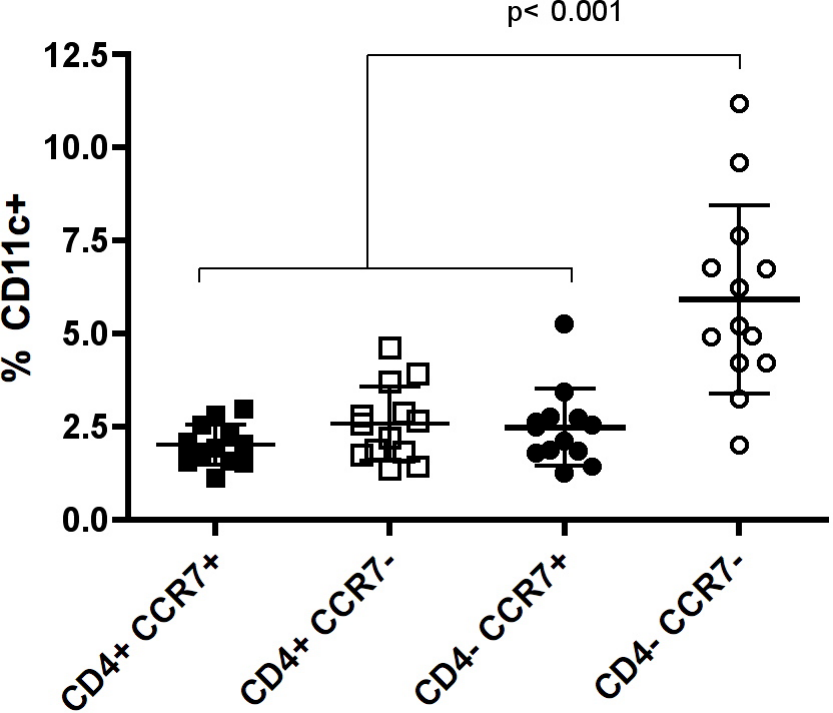
CD11c expression in circulating T cells from healthy young women. CD11c^+^ cells were analyzed by flow cytometry in the CD4^+^ CCR7^+^/^-^ and CD4^-^ CCR7^+^/^-^ CD3^+^ T cell subsets. Gating strategy consisted on the following consecutive gates: lymphocytes, singlets, live CD3^+^ T cells, CD4^+^ or CD4^-^ T cells, CCR7^+^ or CCR7^-^ and lastly CD11c^+^. Each bar represents the mean ± SD of normal donors (n = 13). Data were analyzed by Kruskall-Wallis test with Bonferroni post-test correction.

**Fig 6:**
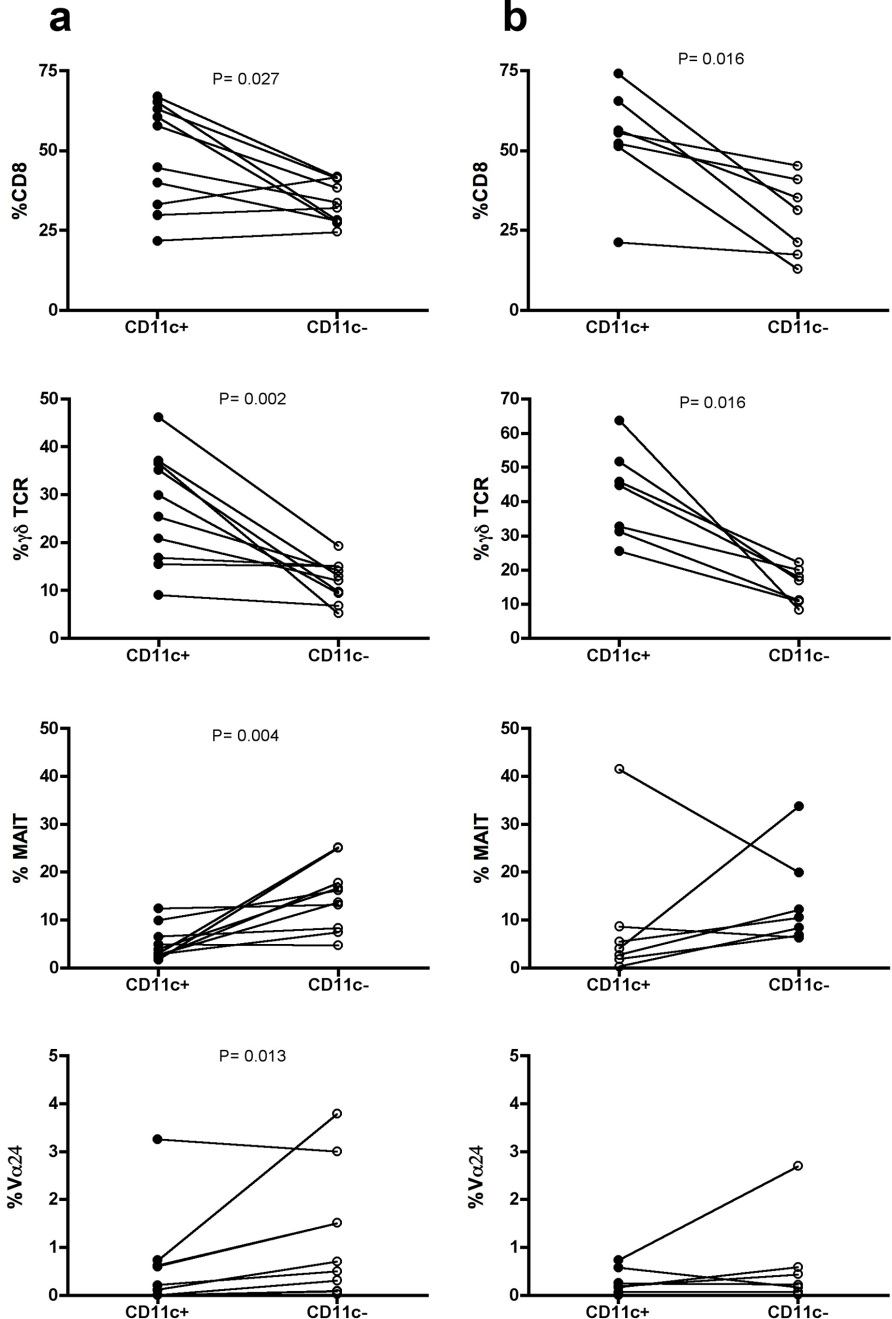
Comparison of specific phenotype frequencies based on CD11c expression in blood samples from healthy women. Comparison of the frequency of CD8, γδTCR, MAIT and iNKT (Vα24) in CD11c^+^ and CD11c^-^, CCR7^-^ CD3^+^ T cells from the same individual. (**a**) Fresh blood (n = 10) and (**b**) PBMC (n = 6). Gating strategy consisted on the following consecutive gates: lymphocytes, singlets, live CD14^-^ CD19^-^ CD3^+^ T cells and CCR7^-^ CD11c^+^or CD11c^-^ T cells (*see* **S3 Fig** *for further details*). Data were analyzed using the paired Student’s t-test.

Next, we examined CD11c expression in CD3^+^ CD4^+^/^-^ and CCR7^+^/^-^ T cells from healthy young women (normal donors, ND), and found an enrichment of this marker in the CCR7^-^ CD4^-^ fraction (**Fig 5**). We then analyzed subsets studied in the mouse model and examined Vα7.2 expression to determine mucosal associated invariant T (MAIT) cells, which are characterized by high CD161 expression (NK1.1 human homolog) [28]. We also adressed iNKT populations via expression of the invariant TCR α chain (Vα24-Jα18). We analyzed each phenotype in circulating CD3^+^ CCR7^-^ T cells, and compared their expression in CD11c^+^ (1.9±0.7%) vs. CD11c^-^ (35.4±10.0%) cells using a non-parametric paired T test (**Fig 6A and S3 Fig left**). The gating strategy used for these analyses excluded CD19^+^ and CD14^+^ cells, which marginally contaminated CD11c^+^ T cells. The only subsets significantly enriched in the positive fraction were CD8^+^ and γδTCR^+^ (**Fig 6A**). In contrast, MAIT cells (Vα7.2^+^ CD161^h^) and iNKT cells were lower in the positive than in the negative fraction (**Fig 6A**). Of note, γδ T cells represented 5.7±2.7% of the total CD3^+^ T cells in blood, and the frequency of CCR7^-^ CD11c^+^ in this subset was of ~10% (**S4A Fig**), from which ~78% were CD161^+^ and ~18% were CD8^+^. In fact, CD161 was similarly high in γδ T cells regardless of CD11c expression (**S4B Fig**), but CD8 expression was doubled in the CCR7^-^CD11c^+^ fraction of γδT cells compared to total CD3^+^ γδTCR^+^ T cells (**S4C Fig**). Regarding CD8^+^ T cells, which represented 28.8±7.7% of the T cells, only about 3.5% of them were CCR7^-^ CD11c^+^ (**S4A Fig**), in which CD161 was enhanced compared to total CD3^+^ CD8^+^ T cells (**S4D Fig**).

In six of these donors, we analyzed the expression of these markers after obtaining PBMC to determine if this process affected any of the T cell phenotypes determined. The overall differences between CD11c^+^ and CD11c^-^ T_EM_ cells were mostly maintained, yet MAIT and iNKT significances were lost (**Fig 6B and S3 Fig right**). Thus, circulating CD11c^+^ T_EM_ cells represented at least two different populations in healthy women: a subset of γδ T cells with their constitutive high expression of CD161 and enriched CD8 expression, and a subset of CD8^+^ T cells with enhanced CD161, yet not belonging to the MAIT or iNKT lineages.

### Human cervical tissue contains γδ T cells expressing CD11c

**Fig 7:**
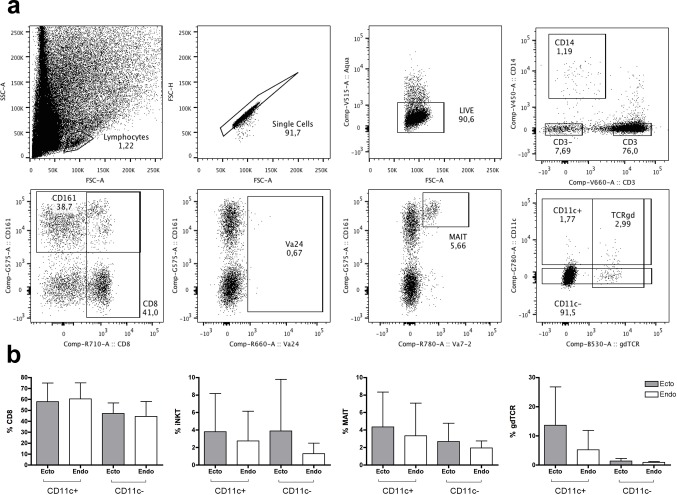
Phenotype of cervically derived T cells from healthy women and analysis by CD11c expression. (**a**) Representative dot plots from the T cell subsets extracted from the endocervix of healthy women. Top row shows the consecutive general gating strategy to select CD3^+^ T cells. Bottom row shows different subsets analyzed in the total CD3^+^ T cells and in the CD11c^+^ and CD11c^-^ T cell fractions. (**b**) Frequency of CD8, iNKT (Vα24), MAIT, and γδT cells by CD11c expression in T cells obtained from cervical tissue. Each bar represents the mean ± SD of the ectocervix and endocervix of each donor (n = 5). Data were analyzed using Wilcoxon matched-paired signed-ranked test.

The same subsets were determined by flow cytometry in cervical samples from healthy women. First, we evaluated the impact of tissue digestion on the detection of these phenotypes in PBMC, since it has been reported that other markers are affected by collagenase treatment [29]. While we confirmed the loss of CD56 expression, we also detected a dramatic reduction on CD19, CD20 and CCR7 protein expression. However, expression of CD3/CD8/CD14/CD16/CD161/αβTCR/γδTCR/Vα7.2/Vα24/CD11c/HLA-DR was not modified. Thus, we analyzed the same subsets without considering CCR7 expression, since most CD3^+^ T cells in these tissues were CCR7^-^ (>95%). Following the gating strategy shown in **Fig 7A**, CD11c expression on CD3^+^ CD14^-^ T cells represented an average of 3.3±1.5% for ectocervix and 3.7±2.5% for endocervix. Since there were no differences between any of the subsets analyzed in the ecto and endocervix (**Fig 7B**), we pooled both tissues in order to strengthen the statistical power of the analysis. This way, the proportion of γδTCR was of >9% in CD11c^+^ vs. ~1% in CD11c^-^ and the proportion of CD8 was of 59% in CD11c^+^ vs. 46% in CD11c^-^ (p<0.006 for both). In three of these samples, we also analyzed the activation marker CD69, which expression was of 86.0±9.5% in CD11c^+^ vs. 74.5±10.1% in CD11c^-^ (**S5A Fig**). Finally, the analysis of the frequency of CD103 in another set of five samples demonstrated no differences based on CD11c expression (**S5B Fig**).

Compared to blood, γδTCR^+^ and CD161^+^ represented smaller populations of total CD3^+^ T cells in the cervix, while CD8^+^ T cells were clearly enriched in this tissue. The mean frequency of CD11c^+^ in γδ T cells was of over 20% (**S4E Fig**), from which CD161 was again independent of CD11c expression (**S4F Fig**), while CD8 was significantly enriched compared to total CD3^+^ γδTCR^+^ T cells (**S4G Fig**). Finally, CD11c^+^ represented 5.8±2.9% of total CD8^+^ T cells (**S4E Fig**), in which CD161 was also significantly different from total CD3^+^ CD8^+^ T cells (**S4H Fig**). Regardless of differences in the percentage of CD8 and other subsets between blood and mucosa in women, similar findings were observed concerning the subsets enriched in CD11c^+^ T cells.

### CD11c^+^ T cells are highly enriched for adhesion molecules expression but not CD103 in women

**Fig 8:**
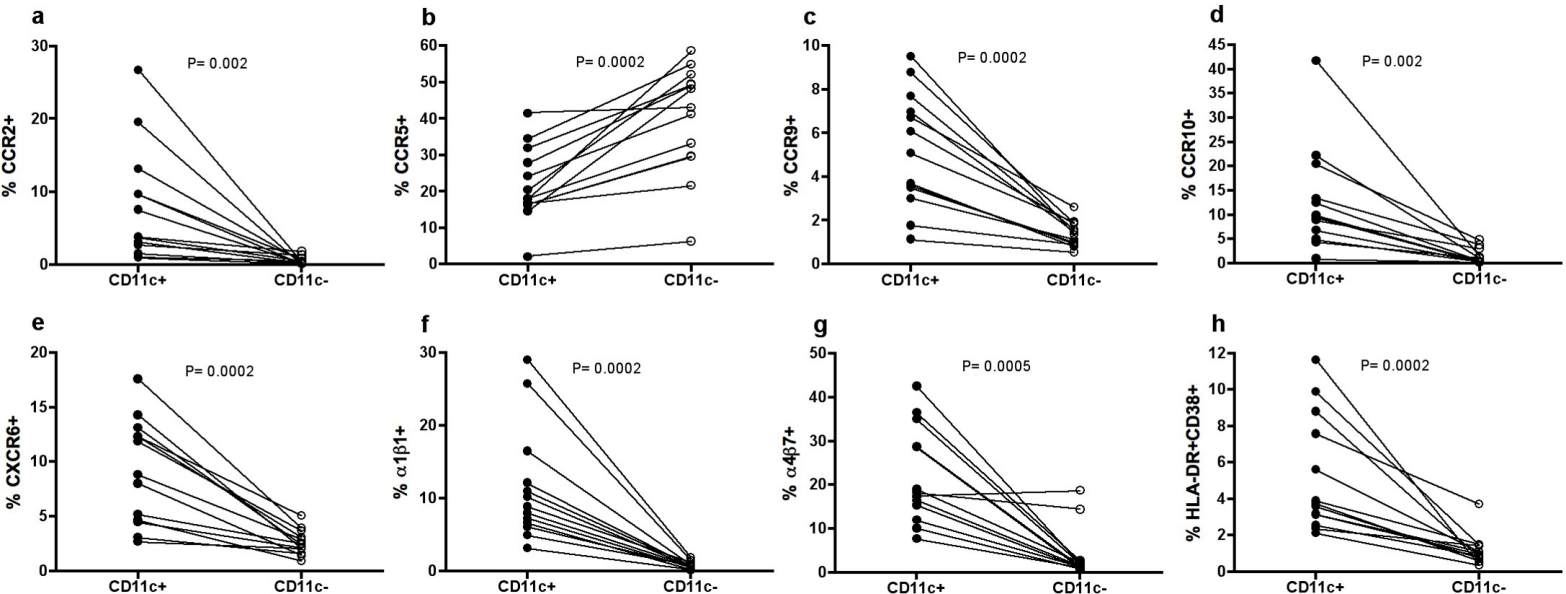
Comparison of adhesion molecule frequencies based on CD11c expression in CD4^-^ T_EM_ cells from healthy women. Comparison of the frequency of (**a**) CCR2, (**b**) CCR5, (**c**) CCR9, (**d**) CCR10, (**e**) CXCR6, (**f**) α1β1, (**g**) α4β7, (**h**) HLA-DR^+^ CD38^+^ in CD11c^+^ and CD11c^-^ CD4^-^ CCR7^-^ T cells from the same individual (n = 13). Gating strategy consisted on the following consecutive gates: lymphocytes, singlets, live CD3^+^ T cells, CCR7^-^ CD11c^+^or CD11c^-^ T cells, CD4^-^ T cells and expression of the different molecules addressed. Data were analyzed using Wilcoxon matched-paired signed-ranked test.

Additionally, we studied the association of CD11c expression with a variety of cellular adhesion molecules in thirteen ND from a previous homing study *(J*. *Qualai et al*., *submitted for publication)*. We observed that many of these molecules were enriched for the CD11c-positive fraction of circulating CCR7^-^ CD4^-^ T cells: CCR2, CCR9, CCR10, CXCR6, α1β1, α4β7 (**Fig 8A and 8C–8G**), as well as CXCR3 (18.7±2.9% vs. 4.5±1.89) and α4β1 (37.4±5.2% vs. 4.5±1.2%) (p = 0.0002; data not shown). In contrast, expression of CCR5 was higher in the negative fraction compared to the positive (**Fig 8B**), while cutaneous lymphocyte antigen and CD103 were not different between these two subsets (data not shown). Additionally, activation markers HLA-DR^+^ and/or CD38^+^ were also significantly increased in CD11c^+^ compared to CD11c^-^ T cells (all p≤0.0006; **Fig 8H** and data not shown). Thus, compared to the animal model, CD103 was not particularly expressed in CD11c^+^ T cells. Still, and similarly to the mouse data, CCR10 was enriched in CD11c^+^ T_EM_ cells, as occurred for most of the other chemokine receptors and other adhesion molecules measured, except for CCR5. This suggests that subsets of T cells expressing CD11c^+^ are activated T cells with high homing potential to a variety of tissues and mucosal compartments.

### CD11c expression in γδ T cells is associated with higher IFNγ secretion after activation

**Fig 9:**
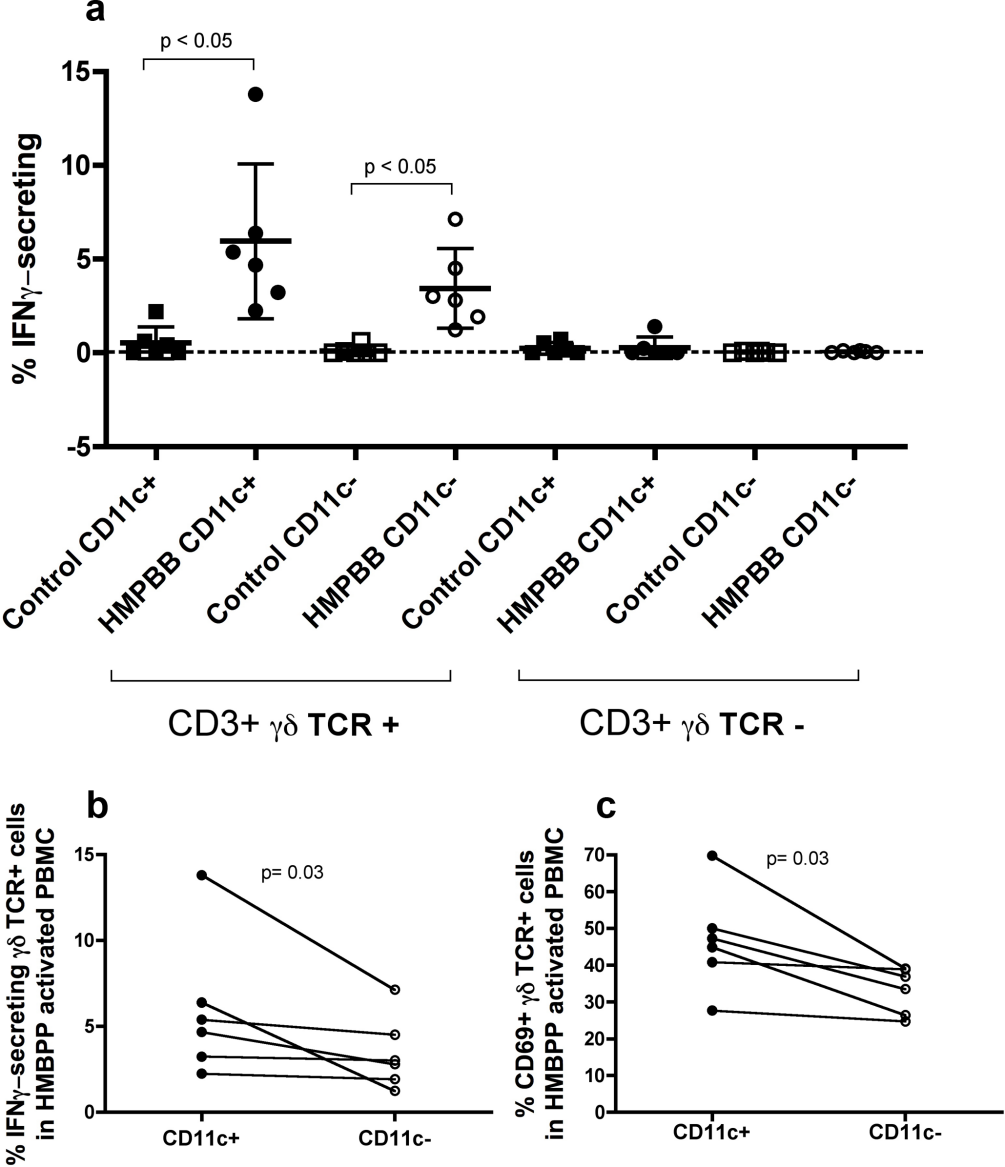
IFN-γ-secreting γδ T cells in PBMC from healthy women after HMBPP activation. (**a**) Comparison of the frequency of γδTCR^+^ CD11c^+^/^-^ and γδTCR^-^ CD11c^+^/^-^ T cells that secrete IFNγ in PBMC from the same individual (n = 6) after 20 hours of (E)-4-hydroxy-3-methyl-but-2-enyl pyrophosphate (HMBPP) activation. Gating strategy consisted on the following consecutive gates: lymphocytes, singlets, CD3^+^ T cells, γδTCR^+^/^-^, CD11c^+^/^-^ and IFNγ/CD69^+^ expression. Each bar represents the mean ± SD of control (squares) and HMBPP-stimulated PBMC (circles) samples. Data were analyzed using the non-parametric Friedman test for repeated measures, with Dunn’s multiple comparisons post-hoc test. Graphs below show the comparison in the frequency of (**b**) IFN-γ-secreting γδ T cells and (**c**) CD69 expression in γδ T cells based on CD11c expression in HMBPP-activated PBMC from the same individual (n = 6). Data were analyzed using Wilcoxon matched-paired signed-ranked test.

CD11c expression in T cells has been associated with multiple effector functions, such as increased IFN-γ secretion [9, 12]. In order to address if activated γδ T cells also differ in their capacity to secrete IFN-γ based on CD11c expression, we stimulated PBMC for 18 hours with HMBPP and then determined IFN-γ secretion and activation. HMBPP is an intermediate of the 2-C-methyl-D-erythritol-4-phosphate pathway of isoprenoid biosynthesis used by many pathogens, and represents the most potent stimulant known of the major γδ T cell population in human peripheral blood [30]. No IFN-γ secretion was detected in conventional T cells or control samples, while 5.9±4.1% of the CD11c^+^ and 3.4 ±2.1% of the CD11c^-^ γδ T cells secreted IFN-γ after stimulation (**Fig 9A**). When samples from the same individual were compared based on CD11c expression we detected significant differences in both, IFN-γ secretion and activation of γδ T cells based on this marker (**Fig 9B and 9C**).

## Discussion

We have attempted to characterize the cellular subsets found in murine and human circulating and GT T cells marked by CD11c expression. While NK1.1 and CD103 expression was tightly associated with CD11c expression in T cells from female mice, these markers were not common in CD11c^+^ T cells isolated from women. Furthermore, although both iNKT and γδ T cell populations were enriched in this fraction in the blood and GT of mice, only the second subset was consistently found in CD11c^+^ T cells from women. These cells are highly activated, express high levels of adhesion molecules, and secrete higher levels of IFN-γ upon activation, when compared to CD11c^-^ T cells. Thus, considering that CD11c^+^ T cells increase after infection or vaccination in different mouse models [9, 12], but also during symptomatic vaginosis *(J*. *Qualai et al*., *submitted for publication)*, we need to reconsider CD11c^+^ T cells in the context of mucosal immune responses.

In murine models of viral infection and graft-versus-host disease, TCR stimulation induces CD11c up-regulation in CD8^+^ T cells [14, 16]. CD11c expression has been associated with gain of effector function, identification of antigen-specific T cells during infection or vaccination (9, 12), and specific T_regs_ [16]. Protection provided by these cells derives from high IFN-γ secretion and up-regulation of effector mechanisms (i.e. granzyme), which account for both effector and regulatory functions [9, 16]. Gene expression analyses of activated CD11c^+^ T_EM_ cells revealed up-regulation of some of these effector mechanisms to be highly related to NK properties [20, 31]. Our data show a clear association between CD11c and NK1.1 expression, which strikingly increases after vaginal infection in mice. Although expression of this molecule on T cells was first employed to exclusively define NKT cells [32], it is now clear that other subsets of non-conventional and activated T cells express NK1.1 or CD161 in mice and humans, respectively [28]. In mice, two different genes encoding proteins with opposite functions share the NK1.1 epitope, namely *Klrb1b* and *Klrb1c* [33]. According to the gene expression analyses we performed, only *Klrb1b* was significantly up-regulated in blood, which would suggest an inhibitory function, although this was not confirmed. However, γδ T cells expressing NK1.1 are the main IFNγ-producers among γδ T cell populations [12] and, as occurred for other NK markers addressed here, differences in the expression of activator (i.e. NKp46) or inhibitory (NKG2A) molecules between blood and tissue could also exist for this epitope. Similarly, in humans, CD161^+^ γδ T cells are efficient producers of IFN-γ but not of interleukin (IL)-17A [34]. In healthy women, we also detected an enrichment of CD161 expression in CD11c^+^ CD8^+^ T cells from blood and cervix; while in γδ T cells, this marker was already high. Although the role of this molecule as inhibitory or co-stimulatory has not yet been fully defined, CD161^+^ T cells, including MAIT and CD161^+^ γδ T cells, have been shown to commonly respond in an innate-like manner to IL-12/IL-18 stimulation independent of TCR activation [28].

Several potential mechanisms could also contribute to the expression of CD11c on T cells. In particular, protein or RNA transfer from activated antigen presenting cells via trogocytosis or external vesicles/exosomes during infection have been described [35, 36]. Although further research should clarify the role of these processes here, the fact that these differences exist during baseline physiological conditions suggest constitutive expression of CD11c by T cells, as others have observed [7]. From all the T cell-subsets analyzed, besides CD8^+^ T cells, the most consistent population expressing CD11c was γδTCR^+^. Indeed, one of the genes that differentiates γδ T cells from other non-conventional T cell subsets is *Itgax*, which encodes the αX integrin [20]. About 10 years ago, Brandes et al. described antigen presenting cell functions by γδ T cells in humans [37]. These cells have signs of pre-activation (CD69^+^) along with up-regulation of a variety of co-stimulatory and adhesion molecules (including HLA-DR and CD11c) [37]. Similarly we found that γδ T cells expressing CD11c are more activated, while CD11c^+^ CD3^+^ T_EM_ cells expressed increased levels of HLA-DR/CD38 and of most of the cellular adhesion molecules addressed compared to CD11c^-^ T cells.

In human blood, γδ T cells and specifically Vγ2Vδ2^+^ T cells, quickly expand after infection in response to microbial metabolites [31, 37]. Thus, we could expect an increase on these cells in the context of GT disorders caused by *Chlamydia* or *Gardnerella spp*. due to production of these metabolites [30]. Although we have not yet been able to confirm if the increment of CD11c^+^ T cells during bacterial vaginosis is due to an increase on this subset, CD11c expression increased in circulating γδ T cells of *Chlamydia*-infected mice, which demonstrated expansion of CD11c^+^ NK1.1^+^ γδ T cells in blood seven days after infection (**Fig 3**). Moreover, CD11c^+^ and γδTCR^+^ cells also increased in the GT after infection. The role of γδ T cells during *C*. *trachomatis* infection was examined in a knockout model of pneumonia by Rank´s group [38]. Although these cells were protective during the first 3–7 days post-infection, they were potentially deleterious at later stages [38]. As suggested, the diversity of their cytotoxic and regulatory effects may impact differently on the course of various inflammatory processes [38]. Additionally, based on the level of CD11c expression, a regulatory (CD11c^high^) and an effector (CD11c^low^) subset have been described for CD11c^+^ CD8^+^ T cells in mice [8]. Future investigations should elucidate if all CD11c^+^ T cell subsets, including γδ T cells, can be similarly distinguished, even in humans.

Further, there was a clear association between CD103 and CD11c expression in mice. CD103 is an αE integrin induced by transforming growth factor (TGF)-β necessary for tissue retention [31, 39]. Interestingly, inducible TGF-β was up-regulated >3–5 times in CD11c^+^ T cells (**Table 1**). CD103 expression, together with the absence of CCR7 and expression of CD69, is one of the hallmarks of tissue-resident memory (T_RM_) CD8^+^ T cells [39]. In naïve mice, only T cells expressing CD11c expressed CD103 in blood and GT, and this association was maintained after infection. Therefore, CD11c could potentially be another marker for T_RM_ in mice. Although no differences were observed in the frequency of CD103 expression based on CD11c expression in the cervix of women, CD11c^+^ T cells in this tissue may also contain CD8^+^ T_RM_ cells, since not all T_RM_ cells express CD103^+^ [40]. Undoubtedly, the relationship between these different phenotypes needs further clarification.

Intraepithelial CD103^+^ CD11c^+^ T cells with an activated phenotype that mediate inflammatory gut pathology during infection have also been described in mice [13]. These cells, composed of ~50% γδ T and αβ T cells, depend on CCR2 for recruitment and, actually, CD11c^+^ T cells express higher levels of CCR2 than CD11c^-^ T cells [13]. In other models, expression of adhesion molecules such as α4β1 on γδ T cells control trafficking into tissue [41]. We determined CCR2 and α4β1 expression in CCR7^-^ CD4^-^ T cells, among other adhesion molecules, and found that during physiological conditions CD11c^+^ are almost exclusively associated with expression of most of these molecules. Likewise, CD161^++^ CD8^+^ T cells, including different unconventional phenotypes, have been proposed to possess critical functions in diverse tissues because of their specific tissue-homing expression patterns [42].

In summary, several animal models evidence that CD11c^+^ CD8^+^/CD11c^+^ NK1.1^+^ T cells are functionally more potent than the ones not expressing CD11c [7, 9–12]. Following respiratory syncytial virus infection, only CD11c^+^ CD8^+^ T cells show signs of recent activation, including up-regulation of CD11b/CD69, and are recruited preferentially to the lung [7]. The characterization of the major subsets included in CD11c^+^ T cells during physiological and infection conditions performed here may recapitulate similar findings described in the literature for these related phenotypes. In humans, this may include the recent description of αβ T cells with DC properties [17], as reported before for γδ T cells [37], and possibly even CD8^+^ T_RM_ [39, 40]. Further research on the role of the different subsets included in this phenotype during mucosal infection is warranted.

## Supporting Information

S1 Fig Significant gene set enrichment of NK biomarkers among differentially up-regulated genes in CD11c positive versus CD11c negative cells. A signal-to-noise ratio (SNR) statistic was computed by GSEA software for each gene in a gene set compared to the rank list of the genes assayed on the microarray ranked according to their correlation with CD11c^+^, this means positively correlated with log2 expression ratios. Significantly enriched gene sets cluster in the up-regulated end of the ranked list have positive enrichment scores of the gene set used for the comparison (red). The graph on the bottom of each panel represents the non-redundant list of genes ranked by differential gene expression between the CD11c^+^ and CD11c^-^ T cells. On each panel, the vertical black lines indicate the position of each of the genes of the studied in the gene set of interest within the rank ordered, non-redundant data set. The green curve corresponds to the ES (enrichment score) curve, which is the running sum of the weighted enrichment score generated by the GSEA software. Shown below are the normalized enrichment scores (NES) for each plot, which are equivalent to the value of the ES curve at the leading edge of the curve (where the statistic reaches its maximum value for a particular gene set). Results show that genes up-regulated in CD11c^+^ cells are significantly enriched for all four gene sets, as judged by the density of hits (black vertical bars) localized at the tip of the blue region with p<0.05 and false discovery rate (FDR) <0.25, but show more significant enrichment in γδ T and iNKT CD4^+^ than in iNKT CD4^-^ cells.
(TIF)

S2 Fig Flow cytometry gating strategy in T cells from peripheral blood and genital tract based on CD11c expression. Representative dot plots showing the frequency of CD11c^+^ in CD3^+^ T cells of: (**a**) peripheral blood of a control animal, (**b**) peripheral blood and (**c**) genital tract (GT) of a vaginally (VAG)-infected animal. For each of these subsets (CD11c^+^ top row, CD11c^-^ bottom row) expression of TCRγδ and CD8α or NK1.1 and CD103 is shown.
(TIF)

S3 Fig Gating strategy of specific T cell phenotypes by CD11c expression in blood and PBMC from healthy women. Example of the frequency of CD161, CD8, Vα24, MAIT and γδTCR in the CD11c^+^ and CD11c^-^, CCR7^-^ CD3^+^ T cell fractions on fresh blood (left) and processed PBMC (right) from the same individual.
(TIF)

S4 Fig γδTCR^+^ and CD8^+^ T cells phenotype based on CD11c expression in blood and genital tract from healthy women. The percentage of CD11c in γδTCR^+^ and CD8^+^ T cells is shown for blood (**a**) and genital tract (GT) (**e**) from healthy women. A comparison on the expression of CD161 and CD8 in CD11c^+^ γδ T cells vs. total γδ T cells from blood (**b and c**) and genital tract (**f and g**) is shown. A comparison on the expression of CD161 in CD11c^+^ CD8^+^ T cells vs. total CD8^+^ T cells from blood (**d**) and genital tract (**h**) is shown. Data were analyzed using Wilcoxon matched-paired signed-ranked test.
(TIF)

S5 Fig Expression of CD69 and CD103 in cervix from healthy women and analysis by CD11c expression. The frequency of CD69 (**a**) and CD103 (**b**) by CD11c expression in T cells obtained from cervical tissue is shown. Each bar represents the mean ± SD of the ectocervix and endocervix of each donor (n = 3–5). A comparison on the frequency of CD69 (**c**) or CD103 (**d**) in T cells from the same individual based on CD11c expression is shown for both cervical tissues. Data were analyzed using Wilcoxon matched-paired signed-ranked test.
(TIF)
