# Supplementary figures and images for: Expression of CD11c Is Associated with Unconventional Activated T Cell Subsets with High Migratory Potential
Source: PLoS One. 2016 Apr 27;11(4):e0154253. doi: 10.1371/journal.pone.0154253 (PMC4847787; doi:10.1371/journal.pone.0154253)

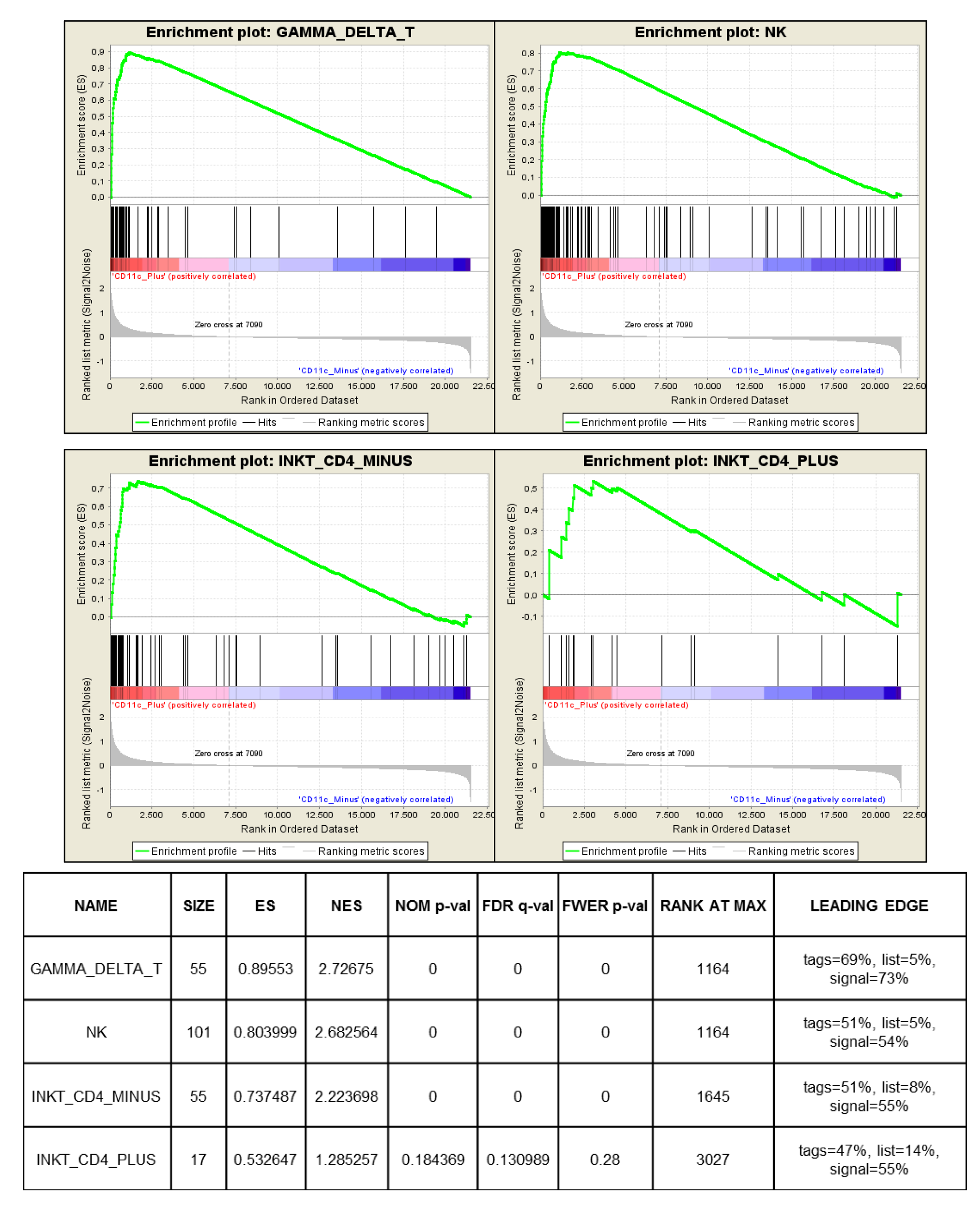

Supplement: S1 Fig — A signal-to-noise ratio (SNR) statistic was computed by GSEA software for each gene in a gene set compared to the rank list of the genes assayed on the microarray ranked according to their correlation with CD11c+, this means positively correlated with log2 expression ratios. Significantly enriched gene sets cluster in the up-regulated end of the ranked list have positive enrichment scores of the gene set used for the comparison (red). The graph on the bottom of each panel represents the non-redundant list of genes ranked by differential gene expression between the CD11c+ and CD11c- T cells. On each panel, the vertical black lines indicate the position of each of the genes of the studied in the gene set of interest within the rank ordered, non-redundant data set. The green curve corresponds to the ES (enrichment score) curve, which is the running sum of the weighted enrichment score generated by the GSEA software. Shown below are the normalized enrichment scores (NES) for each plot, which are equivalent to the value of the ES curve at the leading edge of the curve (where the statistic reaches its maximum value for a particular gene set). Results show that genes up-regulated in CD11c+ cells are significantly enriched for all four gene sets, as judged by the density of hits (black vertical bars) localized at the tip of the blue region with p<0.05 and false discovery rate (FDR) <0.25, but show more significant enrichment in γδ T and iNKT CD4+ than in iNKT CD4- cells. (TIF) [file pone.0154253.s001.tif]

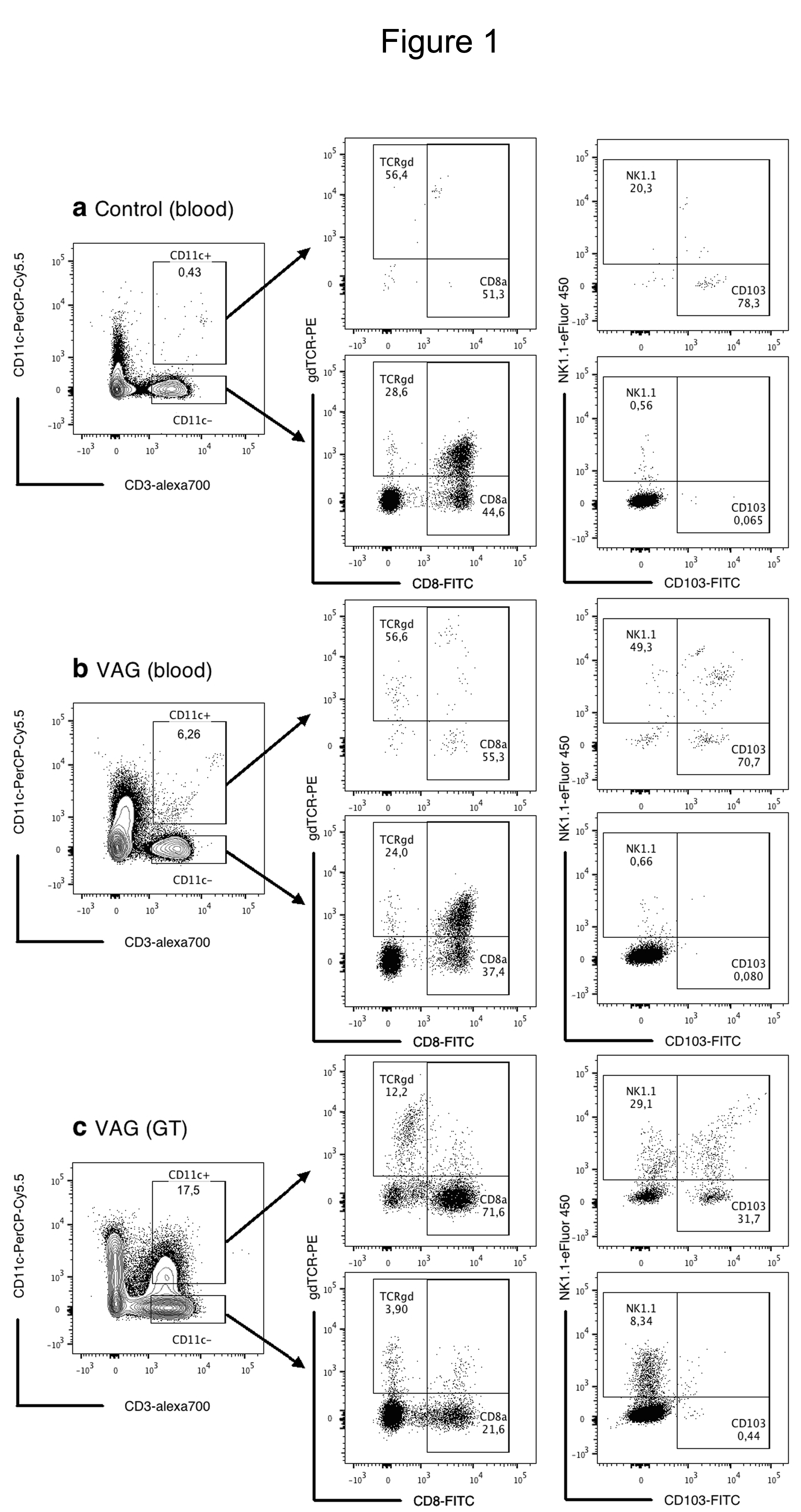

Supplement: S2 Fig — Representative dot plots showing the frequency of CD11c+ in CD3+ T cells of: (a) peripheral blood of a control animal, (b) peripheral blood and (c) genital tract (GT) of a vaginally (VAG)-infected animal. For each of these subsets (CD11c+ top row, CD11c- bottom row) expression of TCRγδ and CD8α or NK1.1 and CD103 is shown. (TIF) [file pone.0154253.s002.tif]

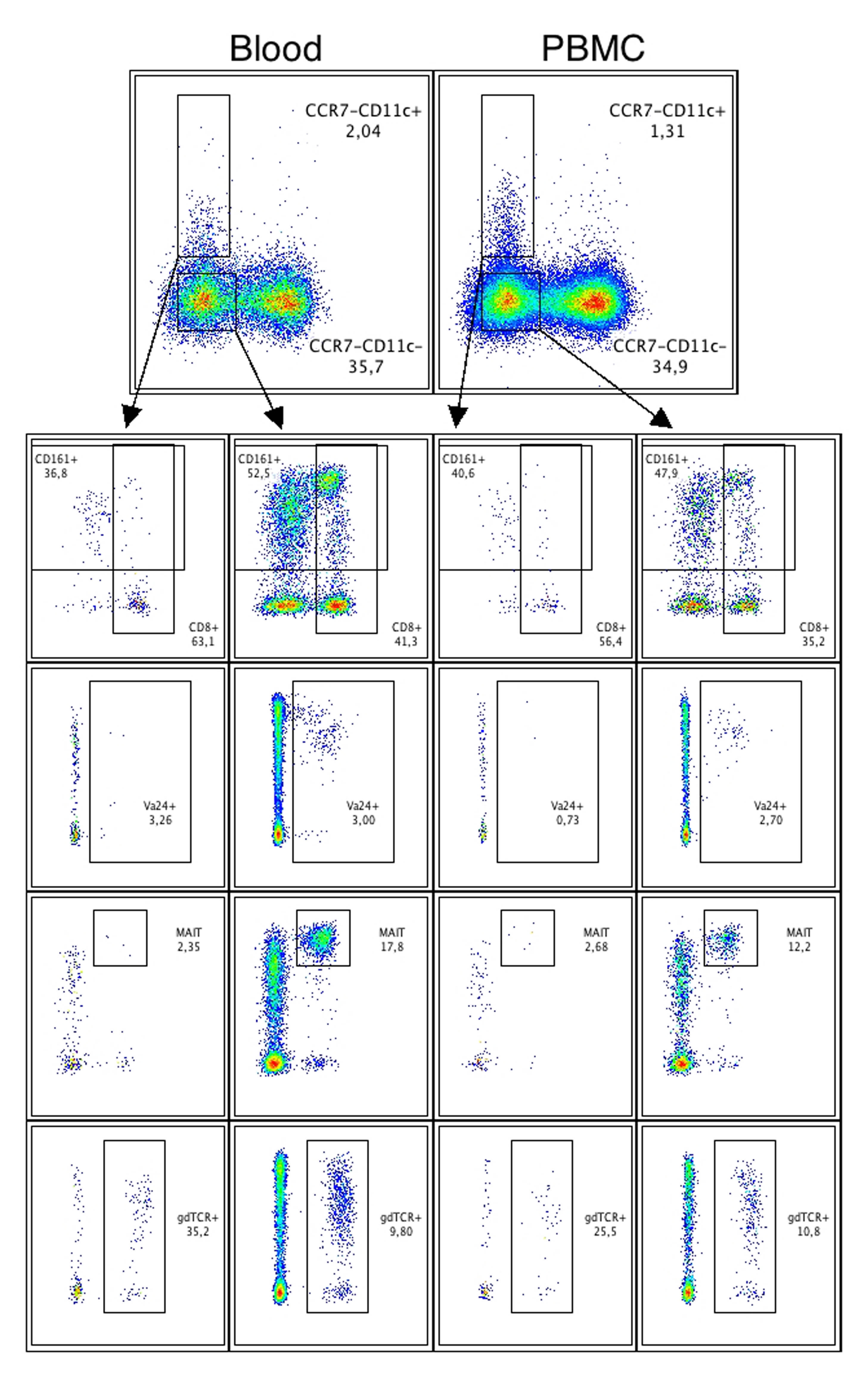

Supplement: S3 Fig — Example of the frequency of CD161, CD8, Vα24, MAIT and γδTCR in the CD11c+ and CD11c-, CCR7- CD3+ T cell fractions on fresh blood (left) and processed PBMC (right) from the same individual. (TIF) [file pone.0154253.s003.tif]
